# Analysis of enterotoxigenic *Bacillus cereus* strains from dried foods using whole genome sequencing, multi-locus sequence analysis and toxin gene prevalence and distribution using endpoint PCR analysis

**DOI:** 10.1016/j.ijfoodmicro.2018.06.016

**Published:** 2018-06-27

**Authors:** Laurenda Carter, Hannah R. Chase, Charles M. Gieseker, Nicholas R. Hasbrouck, Cynthia B. Stine, Ashraf Khan, Laura J. Ewing-Peeples, Ben D. Tall, Gopal R. Gopinath

**Affiliations:** a U. S. Food and Drug Administration, Center for Food Safety and Applied Nutrition, Office of Applied Research and Safety Assessment, Laurel, MD 20708 USA; b U. S. Food and Drug Administration, Center for Veterinary Medicine, Office of Research, Laurel, MD 20708, USA; c Division of Microbiology, National Center for Toxicological Research, Jefferson, AR 72079, USA

**Keywords:** Enterotoxigenic *Bacillus cereus*, 25-gene MLSA, WGS, Genomic characterization

## Abstract

*Bacillus cereus* strains were isolated from dried foods, which included international brands of spices from South East Asia, Mexico and India purchased from several retail stores, samples of powdered infant formula (PIF), medicated fish feed and dietary supplements. The genetic diversity of 64 strains from spices and PIF was determined using a multiplex endpoint PCR assay designed to identify hemolysin BL, nonhemolytic enterotoxin, cytotoxin K, and enterotoxin FM toxin genes. Thirteen different *B. cereus* toxigenic gene patterns or profiles were identified among the strains. Randomly selected *B. cereus* strains were sequenced and compared with reference Genomic Groups from National Center Biotechnology Information using bioinformatics tools. A comprehensive multi-loci sequence analysis (MLSA) was designed using alleles from 25 known MLST genes specifically tailored for use with whole genome assemblies. A cohort of representative genomes of strains from a few FDA regulated commodities like dry foods and medicated fish feed was used to demonstrate the utility of the 25-MLSA approach for rapid clustering and identification of Genome Groups. The analysis clustered the strains from medicated fish feed, dry foods, and dietary supplements into phylogenetically-related groups. 25-MLSA also pointed to a greater diversity of *B. cereus* strains from foods and feed than previously recognized. Our integrated approach of toxin gene PCR, and to our knowledge, whole genome sequencing (WGS) based sequence analysis, may be the first of its kind that demonstrates enterotoxigenic potential and genomic diversity in parallel.

## 1. Introduction

*Bacillus cereus,* a foodborne spore-forming bacterium is a wide-spread human pathogen ubiquitous in the natural environment and found in several categories of food products. It has long been established that *B. cereus* can cause two types of gastrointestinal diseases, i.e., diarrheal and emetic syndromes, which result from the production of various toxins (Blakey and Priest, 1980; Ehling-Schulz et al., 2004). Both diseases can have mild clinical presentations and in most cases, are self-limiting which has contributed to under reporting (Ceuppens et al., 2013).

The virulence of *B. cereus*, whether intestinal or non-intestinal, is intimately associated with the production of tissue destructive/reactive proteins (Bottone, 2010). Diarrhea is caused by enterotoxins produced after ingestion of spores and/or vegetative cells, germination of spores and growth of vegetative cells in the small intestine.

Pore-forming enterotoxins: tripartite hbl enterotoxin hemolysin BL (hbl), nonhemolytic enterotoxin (nhe), a single protein enterotoxin cytotoxin K (CytK), and enterotoxin FM (entFM), are among the secreted toxins (Lund et al., 2000; Schoeni and Wong, 2005). The hbl enterotoxin, encoded by the operon *hblDAC*, is composed of a binding component and two hemolytic components required to maximize the hemolytic, cytotoxic, and dermo-necrotic activities of hbl (Beecher and Macmillian, 1991; Beecher et al., 1995; Heinriches et al., 1993; Ryan et al., 1997). Another three-component enterotoxin, designated nhe, consisting of nheA, nheB, and nheC, is encoded by the *nheBAC* operon (Granum et al., 1999). CytK is a β-barrel pore-forming toxin encoded by *cytK* (Lund et al., 2000; Ramarao and Sanchis, 2013) and is also widely distributed among strains. The *entFM* gene, known for encoding a putative cell wall peptidase, was cloned from *B. cereus* FM1 (Asano et al., 1997). The prevalence of these toxin genes varies among different *B. cereus* strains (Arnesen et al., 2008; Bartoszewicz et al., 2008; Ngamwongsatit et al., 2008).

The emetic toxin, or cereulide, a small cyclic heat-stable dodecadepsipeptide (Agata et al., 1995), is associated with the emetic syndrome. Some investigators have reported that both emetic and diarrheal outbreaks were linked to the same food and it has been reported that both nhe and cereulide can be produced by the same *B*. *cereus* strain (Kim et al., 2010).

*B. cereus* has been isolated from a variety of food sources including rice, potato, dried dairy products, pasta, meat products, coffee, vegetable sprouts, and spices (Egelezos et al., 2010; Fangio et al., 2010; Portnoy et al., 1976; Rajkovic et al., 2006; Samapundo et al., 2011; Wijnands et al., 2006). The Centers for Disease Control and Prevention (CDC) conducted a study between 2000 and 2008 on pathogens causing US foodborne illnesses, hospitalizations, and deaths. For *B. cereus* the estimated annual illnesses observed were 63,000 cases, annual hospitalizations were 20 cases, but no deaths were reported (Scallan et al., 2011).

Powers et al. (1976) reported that spices used in a large variety of food products contain a high prevalence of *B. cereus* (53%), confirming the role played by these additives as a common source of contamination in many countries including, Nigeria, Mexico, Denmark and France, (Antai, 1988; García et al., 2001; Van Doren et al., 2013). Added to this list are reports from Scotland of contaminated spices associated with imported ethnic foods of Chinese and Indian origin. (Candlish et al., 2001). A survey done in the UK on ready to eat foods, found spices or spice ingredients, used in food production, contained sufficient numbers of *B. cereus* which could potentially cause foodborne disease (Little et al., 2003). *B. cereus* is also known to be a common contaminant of milk (Anderson et al., 1995; Lin et al., 1998; Te Giffel et al., 1997).

Recognizing the intra-diversity found within the genus *Bacillus*, a strategy was developed to characterize enterotoxigenic *B. cereus* strains based on conventional gene-specific PCR assays combined with a comprehensive multi-locus sequence analysis scheme (25-MLSA) derived from extensive whole genome sequencing (WGS). In this study we report the prevalence of distinctly divergent *B. cereus* Group Genome types from different FDA-regulated food sources, medicated fish feed and dietary supplements possessing different enterotoxin gene profiles and genomic backbones.

## 2. Materials and methods

### 2.1. Isolation of B. cereus from spices and powdered infant formula (PIF)

Our analysis involved evaluating product blends from all over the world including spices originating from South East Asian, Mexican and Indian markets. Samples of whole black pepper, ground pepper, black pepper powder, paprika, and sesame seeds, along with PIF were assayed for the presence of *B. cereus*. Briefly, duplicate samples containing 20 g of spice or PIF were homogenized (1:10 w:v) in 180 mL Tryptic Soy Broth (TSB) or Modified Buffered Peptone Water (mBPW) (BBL, Becton Dickinson, Franklin Lakes, NJ) supplemented with polymyxin B (0.0127 mg/mL, final concentration). The 200 mL sample was divided into two 100 mL aliquots and one was heated at 56 °C for 30 min and the second one was not heated prior to plating. Using these 2 different treatment methods we devised 2 additional methods. From the heated samples described above one was directly plated onto agar plates (H-D) while the other was incubated at 30 °C, shaking overnight at 150 rpms prior to plating (H o/n). Likewise, the samples not heated were plated directly (NH-D) or plated after an overnight incubation and were designated NH-o/n.

A 10 μL aliquot from each of the samples representing the 4 treatment methods were plated directly onto Mannitol-egg yolk-polymyxin (MYP) agar (Hardy Diagnostics, Santa Maria, CA) and BACARA chromogenic agar plates (bioMérieux, Hazelwood, MO.) and incubated overnight (o/n) at 30 °C. Presumptive strains possessing typical colony morphologies were confirmed as *B. cereus* by plating onto 5% sheep blood agar (TSAB, Remel, KS) for demonstration of hemolytic activity. Final identities were made following the identification scheme described in the Bacteriological Analytical Manual BAM protocol (Tallent et al., 2012). Two colonies from the same agar plate were picked for analysis. For quality control purposes, *B. cereus* ATCC 14579 was used as a positive control for all plating and PCR experiments and *B. thuringiensis* ATCC 29730 was used as a negative control to differentiate *B. cereus* from *B. thuringiensis*. Frozen stocks were maintained in TSB supplemented with 50% glycerol and stored at −80 °C.

### 2.2. B. cereus isolated from dietary supplements and medicated fish feed

Nine strains from dietary supplement samples were obtained from the National Center for Toxicological Research (NCTR). Foods included supplements: used for weight loss, to enhance athletic performance, and to reduce mental and physical fatigue. (Foster et al., 2013). One additional supplement included in this study was purchased at a local store, and is described as a fat-burner supplement containing chitosan used for weight-loss and to increase energy levels. Take note that the health benefit statements of these dietary supplements as described are based on packaging information and the statements do not reflect endorsement by the FDA.

Twenty-three strains, isolated during a study that used medicated fish feed containing the antibiotic oxytetracycline were obtained from the Center for Veterinary Medicine (CVM), FDA.

Dietary supplement and the medicated fish feed strains were inoculated onto BACARA chromogenic agar plates, and incubated (o/n) at 30 °C. Strains with typical colony morphologies were confirmed as *B. cereus* by plating onto 5% sheep blood agar TSAB for demonstration of hemolytic activity, and by PCR using primers specific for *B. cereus* and *B. thuringiensis* crystal toxin gene (*cry*).

### 2.3. Extraction of DNA from strains

DNA was extracted from *B. cereus* strains grown in 6 mL of TSB and incubated with shaking (180 rpm) at 30 °C o/n. Two mL aliquots of these cultures were pelleted (5000 ×*g* for 10 min.) and the supernatants removed. The cells were then suspended in 180 μL of enzymatic lysis buffer (20 mM Tris-HCl, pH 8.0, 2 mM sodium EDTA, 1.2% Triton X-100) containing 20 mg/mL lysozyme and incubated for 4 h at 37 °C. Ten microliters of RNase A (10 mg/mL) was added to each sample and incubated for 5 min. at room temperature prior to initiating the QIAcube program. Genomic DNA was isolated from the pellets of each of the 2 mL aliquots of the cultures using the robotic QIAcube workstation with its automated Qiagen DNeasy chemistry (Qiagen, Germantown, MD) for purification of DNA, following the manufacturer’s recommendations. Typically, 5–15 ng/μL of purified genomic DNA was recovered in a final elution volume of 200 μL. Genomic DNA was stored at −20 °C until needed. As an alternate method we used an UltraClean microbial DNA isolation kit (MO BIO Laboratories, Carlsbad, CA USA) according to the manufacturer’s instructions for each strain. The DNA, approximately 30–260 ng/μL, was recovered in certified DNA-free Tris buffer and again stored at −20° until needed.

### 2.4. PCR amplification of enterotoxin genes

Enterotoxin gene profiles were determined by using an endpoint PCR assay with primers specific for *hblDAC*, *nheBAC*, *cytK*, and *entFM*. Primer sequences were as published by Choo et al. (2007); Ngamwongsatit et al. (2008); and Thaenthanee et al. (2005). The amplification reactions were carried out in an Applied Biosystems 2720 Thermal cycler (AB, Applied Biosystems, Inc., Foster City, CA). Amplicons were separated on a 1.5% agarose gel using a 1Kb DNA molecular size standard (Invitrogen, Carlsbad, CA) to estimate sizes. *B. cereus* ATCC strain 14579 and *B. thuringiensis* ATCC strain 29730 were used as reference strains. The enterotoxin gene profiles are shown in Table 1.

### 2.5. Whole genome sequencing of B. cereus isolates from food and feeds samples

**Fig. 1.**
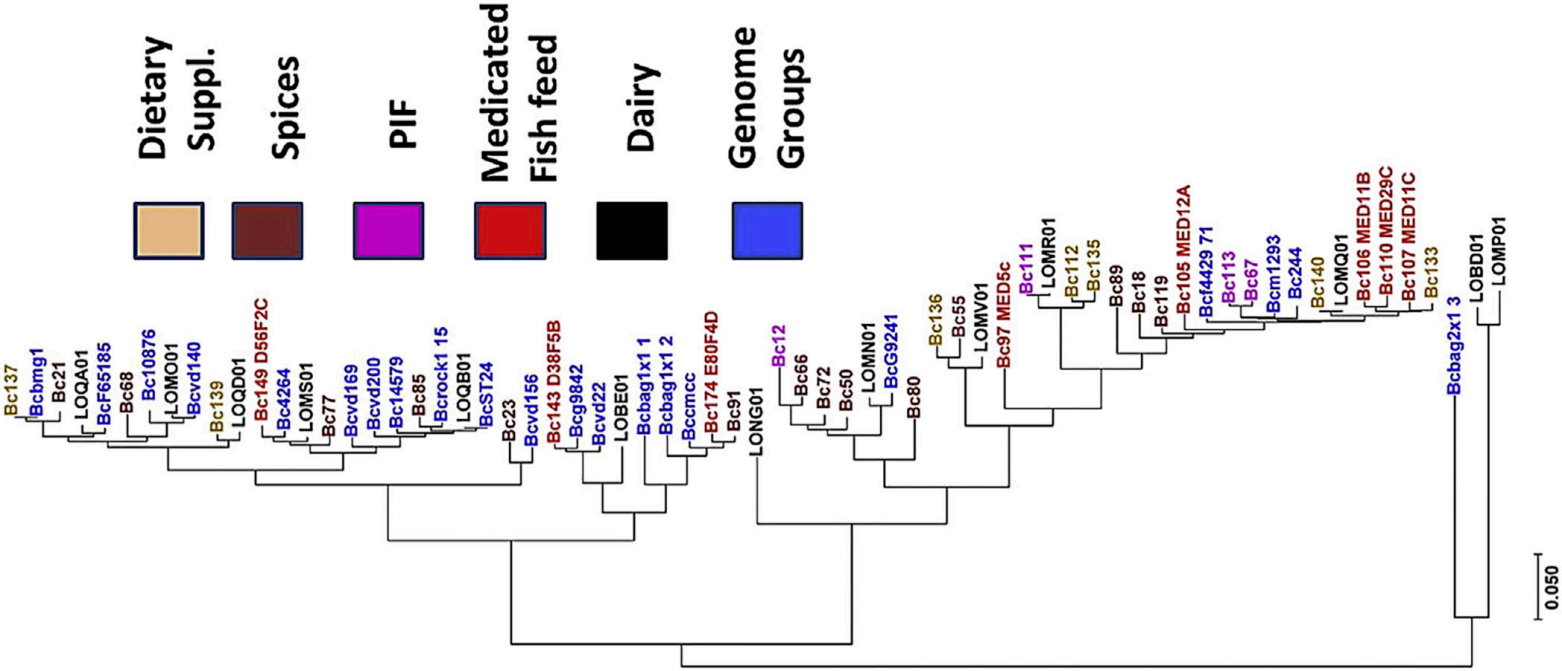
Phylogenetic analysis using the 25-gene MLSA strategy with 66 *B. cereus* genomes. The tree was developed by comparing 25 loci from these genomes with *B. cereus* 14579. A 66-genome data matrix was constructed using these alleles which contained 2179 base positions. This allelic data matrix was then subjected to phylogenetic analysis using the Neighbor-Joining algorithm in MEGA7 (Kumar et al., 2016). The sources of the isolates are color coded.

A total of 66 *B. cereus* genomes were used for comparative genomic analyses. This includes (Fig. 1) thirty-two genomes from the *B. cereus* strains isolated from spices, PIF, dietary supplements, and medicated fish feed samples (this study); genomes from 13 *B. cereus* strains previously recovered from dairy (Kovac et al., 2016), and 21 ‘Genome Groups’ genomes from the National Center Biotechnology Information (NCBI) databases which grouped *B. cereus* genomes by their nucleotide relatedness. At the beginning of this study, these 21 annotated genomes were part of a larger set of Genome Groups designated by NCBI based on nucleotide relatedness among *B. cereus* genomes. When this manuscript was prepared for submission, these Genome Groups were incorporated into a routine phylogenetic analysis to create NCBI Genome Tree reports (https://www.ncbi.nlm.nih.gov/genome/tree/157, last accessed 2/1/2018). The GenBank sequences listed in the Table 2 were part of the original NCBI Genome Groups. Library preparations and WGS were completed for 32 strains from various dried foods, dietary supplements and animal feed using Nextera XT Library Kit and MiSeq platform respectively (Illumina, San Diego, CA, USA). De novo assembly of 500 cycle, paired-end read datasets was routinely carried out using the recommended workflow on CLC Genomics Work bench version 8.0 (CLC bio, Aarhus, Denmark).

### 2.6. Comparative genome analyses of 66 B. cereus genomes

Sequence analysis was completed on all 66 *B. cereus* genomes (Table 2) using different approaches. PCR reactions as described by Priest et al. (2004) were routinely performed to generate MLST loci sequences which were queried against the SuperCAT database (Tourasse and Kolstø, 2008). The known and new sequence types (STs) are tabulated in Table 2. Initially, a local BLAST database of these *B. cereus* genomes (Altschul et al., 1990) was employed for querying using in-house perl scripts.

This seven-gene approach (Priest et al., 2004) was expanded to include additional loci available on the SuperCAT database and derived from the scheme by Helgason et al. (2004), Sorokin et al. (2006), Ko et al. (2004) and Tourasse et al. (2006). Based on these five published schemes outlined in the *B. cereus* SuperCAT database a non-redundant, comprehensive multi-loci sequence typing scheme (25-MLSA) was developed to be used with WGS datasets from this and subsequent studies. Nucleotide sequences of 25 markers in this list were obtained from *B. cereus* 14579 (GenBank Accession (GCA_000007825) as the reference strain. These 25-MLSA loci were used to query the local BLAST database to identify alleles across the genomes. The resulting “25-MLSA”-based allelic data matrix was used for phylogenetic analysis.

*B. cereus* ATCC 14579 was independently annotated using RAST (Aziz et al., 2008), resulting in 5538 coding DNA sequences (CDS). Using the local *B. cereus* genome database, we had created, 4430 out of 5538 *B. cereus* 14579 genes were identified to have homologs in all the 66 genomes and were designated as “conserved genes”. This list of conserved genes was used to identify alleles across the 66 genomes. A large data matrix comprised of thousands of alleles from these 4430 “conserved genes” in these 66 genomes was generated. We randomly selected 1000 loci for phylogenetic analysis.

A whole genome SNP analysis was carried out using the kSNP3 program (Gardner et al., 2015). kSNP3 software generates a unique list of sequences of pre-determined virtual fragments called k-mers (k = random size) from the query genomes. A specific in-build algorithm was applied to determine the optimal k-mer size to be used based on the input genomes. In our genome-wide analysis, a k*-*mer size of 19 was chosen by the kSNP3 program. High quality alleles in these k-mers in all the queried genomes are generated and the resulting data matrix file with these alleles is used for downstream analysis. The resultant “whole genome” SNP data matrix was analyzed for phylogenetic relationship.

The data matrices resulting from the three genome-based analytical methods described above, viz. 25 MLSA, conserved genes-based and whole genome SNP were used for phylogenetic analysis in MEGA7 suite (Kumar et al., 2016). The Neighbor-Joining algorithm was used to build trees with bootstrap values using the utilities provided in MEGA7 suite. Splitstree analysis (Huson and Bryant, 2006) was used to compare phylogenetic relatedness among the strains as identified by 25- MLSA and kSNP3 analyses when needed.

## 3. Results and discussion

### 3.1. Prevalence of B. cereus enterotoxins genes in spices and PIF isolates

Strains were identified by the presence of hemolytic, lecithinase and β-glucosidase activities and absence of the *Bacillus thuringiensis* insecticidal crystal toxin, *cry* gene. Sixty-four *B. cereus* isolates obtained from paprika, several grades of black pepper (whole black pepper, ground black pepper, and black pepper powder), sesame seeds, and PIF were analyzed for enterotoxin gene prevalence and distribution. The enterotoxins primarily associated with foodborne illness and targeted in this study included hemolysin, hbl (*hblDAC)*, nonhemolytic hemolysin, nhe (*nheBAC)*, cytotoxin K (*cytK)*, and enterotoxin FM (*entFM).* The hemolysin *hbl* was the most prevalent toxin gene observed in our isolates (Table 1). In contrast, Hansen and Hendriksen (2001) and Guinebretière et al. (2002) found isolates with only *nhe* present (63%) and isolates with both *nhe* and *hbl* (37%) present in clinical and food-borne *B. cereus* isolates, respectively. In a study by Ankolekar et al. (2009) it was reported that 55 (59%) of 94 isolates produced both the *nhe* and *hbl* toxins, whereas 89 (95%) were positive for either enterotoxin. In our analysis some of the isolates possessed *hblDAC* genes without the presence of the *nheBAC* genes. Interestingly, Granum (2001) reported that the *hbl* operon is only present in about 50% of foodborne *B. cereus* isolates, while *nhe* operon is present in almost 100% of the strains. These findings are supported by similar results described by Arnesen et al. (2008) and Moravek et al. (2006). Together, these results suggest that toxin gene prevalence and distribution among *B. cereus* strains varies greatly. Furthermore, according to Agaisse et al. (1999), Gohar et al. (2002), and Lindbäck et al. (2004), expression of both *hbl* and *nhe* is positively regulated by the pleiotropic regulator PlcR. Later Lindbäck et al. (2010) reported that both nheB and nheC are required for membrane binding and complex formation, and nheA triggers the cytotoxicity. Guinebretière et al. (2010) and Ngamwongsatit et al. (2008) reported that all *B. cereus* strains possess the *nhe* genes, even though highly divergent nucleotide sequence variations may exist. Biochemical and genetic characterization of several similar strains led to a recent designation of a new species, *Bacillus cytotoxicus* (Guinebretière et al., 2013), which features an unusual *nhe* operon, encoding the components of nhe. The role that nhe plays in the virulence of some of these distinct strains is mainly attributed to the greater cytotoxic activity of the pore-forming cytotoxin K (Fagerlund et al., 2004, 2007) which is thought to cause lysis of small intestine epithelial cells, resulting in diarrhea (Hardy et al., 2001; Lund et al., 2000). Gene profile results of the *B. cereus* strains isolated from spices and PIF are summarized in Table 1.

We identified eight different toxin gene profiles (I-VIII) among 51 positive strains isolated from spices and PIF. The most prevalent toxigenic gene profile included the presence of *hblDAC*, *nheBAC*, *cytK* and *EntFM* genes, under profile I. Twenty-one (21/51, 41%) spice and PIF strains were classified in this group. Some of the strains were obtained from different areas of a sample, (i.e., subs, taken from the top, middle or bottom). Ten (20%) of the 51 samples tested possessed *hblDAC* toxin genes. (Profile II).

It should be noted that in some cases, direct plating versus an overnight enrichment, as well as, heating versus not heating of the same sample, led to the isolation of strains with different toxin genotypes. Nonetheless, these results suggest that these samples possessed a mixed population of strains with varying genotypes, supporting the hypothesis proposed by others (Ehling-Schulz and Messelhäusser, 2013) that certain characteristics, i.e. toxin genes, are more strain-specific. These results clearly emphasize the need for further surveillance studies to understand the enterotoxigenic diversity of *B. cereus*.

Toxin gene profile IV included six strains which possessed *nheBAC, cytK* and *entFM* toxin genes. These strains were all recovered from whole black pepper and black pepper powder originating from two different manufacturers. One possible explanation for this could be these two manufacturers bought their raw material from the same supplier. It is interesting to note that many of the strains taken from the whole black pepper samples fell under three different toxigenic profiles and were obtained using various treatment regimens. Six out of seven strains from paprika were positive for *hblDAC* toxin genes (toxin profile II). Strains obtained from the sesame seed samples also showed a diverse toxigenic genotype pattern consisting of six different profiles (I–II and V–VII, Table 1). One sesame seed strain was found to only possess the *nheBAC* gene cluster (toxin profile III). Hansen and Hendriksen (2001) reported that enterotoxin proteins produced by strains in which specific *nhe* or *hbl* genes were not detected may be related to gene sequence polymorphisms. We also observed that many of our strains lacked one or more of these genes.

Toxigenic *B. cereus* was present in several PIF samples as well. The incidence of *B. cereus* spores found in dried milk products, which was recently reported (Merzougui et al., 2014) confirms earlier findings by Bartoszewicz et al., 2008; of high contamination levels of this micro-organism in PIF. The incidence of *B. cereus* in infant food alone has been reported to be as high as 54% of 261 samples distributed in 17 countries. (Becker et al., 1994). Two different gene prevalence patterns emerged from the PIF strains investigated in the present study. Four of the 11 PIF strains (36%) possessed all the toxin genes. Most of the PIF strains however, (7 of the 11 or 64%), had a toxin gene profile that included *hblDAC*, *nheBAC* and *entFM*, but lacked *cytK.*

The finding of *cytK* in the majority of spice, dietary supplements, and medicated feed strains supports the results of Guinebretière et al. (2002) and Oltuszak-Walczak and Walczak (2013) who reported that this gene is widely distributed among strains belonging to several *B. cereus* Genome Groups. Likewise, other reports describe the presence of *cytK* in numerous *Bacillus* species as being more of a strain-specific rather than a species-specific attribute (Ehling-Schulz and Messelhäusser, 2013).

### 3.2. Prevalence of B. cereus enterotoxin genes in dietary supplement strains and medicated fish feed

All the dietary supplement strains had the *nheBAC* toxin gene cluster and *entFM* (representing toxin profiles I, IV and IX) except for one strain lacking the *nheA* toxin gene (toxin profile X). Three strains, out of nine (33%), possessed all the toxin genes, which included *hblDAC*, *nheBAC*, *cytK* and *entFM* (toxin profile I). Only five of the dietary supplement strains carried the *cytK* gene.

A total of 23 medicated fish feed strains were analyzed for the presence of *B. cereus* enterotoxin genes. Among the strains, there were six toxin gene profiles observed (1, IV, IX, XI–XIII). Toxin genes *nheBAC* and *entFM* were present in all the strains.

Sixteen of the 23 strains (70%) were positive for the *cytK* gene. Two strains were positive for both the *hblD* and *hblC* genes, whereas the frequency of having *hblC* gene was 48% among these strains (11 of 23); occurring under 4 different profiles (profiles I, XI, XII, XIII). Notably only 1 medicated fish feed strain was positive for all eight *B. cereus* enterotoxin genes (*hblDAC*, *nheBAC*, *cytK* and *entFM*, profile I).

### 3.3. Phylogenetic analysis and whole genome sequencing

As part of the food safety surveillance program, we routinely analyze food samples for the presence of *B. cereus*. Many of these strains from the *B. cereus* complex have been sequenced as part of the FDA GenomeTrakr project (Allard et al., 2016) and we have included 32 of these genomes in this study. Whole genome assemblies of strains from spices, PIF, dietary supplements and medicated fish feed samples were generated as described. A single local database consisting of genomes from 32 of our strains, 13 dairy genomes (Kovac et al., 2016) and 21 NCBI Genome Groups was used in the subsequent analysis. Table 2 shows the sequence types of 32 of our strains generated using the seven house-keeping genes described by Priest et al. (2004). It has been reported (Ceuppens et al., 2013; Priest et al., 2004) that the *B. cereus* complex has significant nucleotide diversity among strains with expanding allelic information as new genomes are sequenced. In our hands, extensive sampling of diverse food sources has a high potential for finding previously unidentified sequence-types. For example, in our dataset of 32 samples, we describe four new STs while 13 of the strains did not fit any of the known sequence types.

It was not surprising the 7-loci MLST scheme initially used was in-adequate to capture this diversity. At least five MLST schemes have been reported for the *B. cereus* group.

(Tourasse and Kolstø, 2008). SuperCAT database (http://mlstoslo.uio.no/, last accessed 2/1/2018) exploits high powered computing using supertree reconstruction algorithms (Biniinda-Edmonda, 2004) by combining MLST typing from each of these schemes routinely for hundreds of genomes. We adapted this integrated approach in a different way by combining loci from different schemes to type the *B. cereus* isolates from food and feed. This approach is more of an expanded MLST approach with 25 unique and tested housekeeping genes instead of seven. The comprehensive 25-gene MLSA strategy was designed using unique sequences derived from *B. cereus* 14579 genome, as described earlier. With the 66-genome local database, alleles located in 2179 base positions were detected. This allelic data matrix was subject to phylogenetic analysis in MEGA7 and a high-resolution cladogram was generated using Neighbor-Joining algorithm (Supplemental Fig. 1). The bootstrap test scores pointed to the confidence in the cladistics relationship among the strains after testing the reproducibility of the analysis 500 times. Strains from foods and feed clustered with dairy and other Genome Groups intermittently. The larger cluster consisted of 16 of the 21 Genome Groups with only a handful of the genomes from the strains used in this study grouping in this cluster. Most of these clustered in a second clade (bottom clade in the figure) suggesting a deeper intra-species genomic diversity than what has been previously recognized. It is clear from this analysis (Fig. 1) that food and feed strains from this study represent unreported phylogenetically-related clusters within the *B. cereus* Genomic Group species complex. By anchoring unknown strains from our samples against the NCBI Genome Groups, we could compare them with other *B. cereus* strains from many kinds of FDA-regulated commodities like spices, PIF, dietary supplements, dairy and medicated fish feed. Public health agencies like FDA and CDC are relying more on WGS based approaches for typing and source tracking (Allard et al., 2016). The 25-MLSA approach enables rapid typing and identification of *B. cereus* complex isolates before submission to the above GenomeTrakr project for public use. To evaluate the robustness of our comprehensive 25- MLSA approach, we used two different bioinformatic tools. The first approach created a list of 4430 conserved gene sequences from a reference genome (in this case 25 loci based on the *B. cereus* 14579 genome) and contained variant alleles in more than 120,000 positions across the 66 genomes. We randomly selected 1000 genes out of these 4430 query loci alleles for further analysis with MEGA7 suite. The phylogenetic tree from this analysis (Supplemental Fig. 1) showed a high degree of congruency when compared with the phylogenic clusters generated by 25-MLSA (Fig. 1)

The second approach consisted of detecting single nucleotide polymorphisms (SNPs) across the 66 genomes using kSNP3 (Gardner et al., 2015), a widely used whole genome SNP finding tool. This analysis resulted in a large data set of SNPs present in 11,000 positions across 66 genomes which were used to build a similar phylogenetic tree (Supplemental Fig. 2). When the two trees generated from conserved genes and the kSNP3 tools were compared in splitstree, no significant anomalies were observed (data not shown). When compared with the 25-MLSA-based tree as shown in Fig. 1, these two approaches produced highly similar cladograms. It is interesting to note the trees from 79% of the coding genes and the whole genome based k-mers showed indistinguishable phylogenetic relationship among the isolates, and these two trees were highly congruent with the 25-MLSA approach developed for this study.

The results from the two alternative methods confirmed that our approach of using 25-MLSA with 25 housekeeping genes was sufficient to capture the intra-species genomic diversity of *B. cereus* strains. These analyses pointed to a highly similar genomic backbone with significant and comparable intra-species nucleotide divergence deep enough to be captured singly by the 25-MLSA approach described in this work.

The close phylogenetic alignment of Genome Groups in the upper cluster (Fig. 1) also points to previously unrecognized deep intra-species divergence among the food and feed strains. This work highlights the necessity of extensive sequencing of *B. cereus* strains from foods, feed and associated environments to fill this gap. In contrast, nine dairy strains are distributed almost evenly (Fig. 1) within different Genome Groups in the tree.

The elevated level of genetic relatedness among the members of the *B. cereus* complex has been confirmed by comparative genomics (Tourasse et al., 2006). The existence of high levels of synteny and conserved genome sequences point to a closely related group of organisms. By increasing the number of loci from seven or nine to 25 under the 25-MLSA approach described here, the intra-species sequence divergence among the clades of *B. cereus* strains was captured. The result of whole genome SNP profiling using kSNP3 and the alleles from 1000 conserved coding genes highlights the limitations of quantifying nucleotide differences as seen by 7-gene MLST. It is important here to consider plasmid-borne genes and genes associated with mobile elements in the genomes for strain differentiation as suggested previously (Rasko et al. (2005). The genotyping strategy confirmed the presence of the enterotoxin genes, while also elucidating strain-level genotypic differences.

## 4. Conclusion

Our approach, consisting of parallel bioinformatics, comparative genomics and endpoint PCR analyses of enterotoxin genes, represents a comprehensive strategy for characterizing *B. cereus* enterotoxigenic strains from dried foods and feed. The results of our toxigenic profiles have led us to speculate that certain profiles may be specific for certain types of food. Since *B. cereus* enterotoxin genes are known to be strain specific, certain commodities may allow for colonization of particular strains which possess a specific toxin genotype. However, more isolates from these same commodities and other commodities should be studied before this can be definitively answered.

With WGS becoming a routine technique for food safety surveillance studies of cases and outbreaks, the genomic data are now being used extensively for source tracking and identification of foodborne pathogens (Toro et al., 2016; Allard et al., 2016) In the present study, we chose 25 loci used in 5 different MLST schemes that were applied by SuperCAT database for its supertree reconstruction to create a comprehensive 25-MLSA method. This was an expanded MLST-like method necessary to capture emergent and expanding allelic variations among *B. cereus* complex isolates. Predictably, the 25-MLSA based phylogenetic analysis, separated 45 *B. cereus* genomes from spices, PIF, dietary supplements, dairy and medicated animal feed samples into distinct groups that ignored food sources. In addition, this method seamlessly sorted these isolates into 21 anchors that NCBI had identified as genomically distinct groups among the growing number of *B. cereus* genomes (totaling 66). This robust method was further evaluated against two computation-intensive bioinformatic tools for validation.

Thus, we suggest that our 25-MLSA approach provides a simple, but rapid tool, to investigate WGS datasets from uncharacterized *B. cereus* strains for comparison with established genomes. By confirming the high level of similarity in the genomic backbone with specific but discrete intra-species divergence, and combining WGS-based 25-MLSA approach with specific endpoint PCR analysis, our study recognizes the use of *B. cereus* toxin genes as an important component in the toolbox for food safety investigations associated with *B. cereus*.

Whole genome sequencing has become an integral and helpful part of our agency’s routine workflow in responding to foodborne microbial contamination events.

The current approach of analyzing WGS assemblies with our comprehensive 25- MLSA approach could easily be extended to include selected plasmid-borne genes and virulence factors. This study establishes a powerful platform for further genomics research of the phylogenetically diverse *B. cereus* group, a prerequisite towards development of future countermeasures against this important foodborne pathogen.

## Supplementary Material

Supp fig 1

Supplemental 1

Supplemental 2

Supplemental 3

Supp Fig 2

## Figures and Tables

**Table 1:** Gene profiles of enterotoxigenic *B. cereus* strains isolated from spices, PIF, dietary supplements, and medicated fish feed as identified using PCR.

Spice sample	Treatment method	Target gene								Profile	No. of samples in each profile
		hblD	hblA	hblC	nheB	nheA	nheC	cytK	entFM		

Whole black pepper	HD, NH-D, H o/n, NH o/n	(+)	(+)	(+)	(+)	(+)	(+)	(+)	(+)	Profile I	12
	H o/n, NH o/n	(−)	(−)	(−)	(+)	(+)	(+)	(+)	(+)	Profile IV	4
	NH o/n	(−)	(−)	(−)	(−)	(+)	(−)	(+)	(−)	Profile VI	2
Ground black pepper	HD, NH o/n	(+)	(+)	(+)	(+)	(+)	(+)	(+)	(+)	Profile I	4
Black pepper powder	HD	(+)	(+)	(+)	(−)	(−)	(−)	(−)	(−)	Profile II	1
	H o/n, NH o/n	(−)	(−)	(−)	(+)	(+)	(+)	(+)	(+)	Profile IV	2
Paprika	HD, NH-D, H o/n, NH o/n	(+)	(+)	(+)	(−)	(−)	(−)	(−)	(−)	Profile II	6
	H o/n, NH o/n	(−)	(−)	(−)	(−)	(−)	(−)	(−)	(+)	Profile VII	1
Sesame seed	NH o/n	(+)	(+)	(+)	(+)	(+)	(+)	(+)	(+)	Profile I	1
	NH-D, H o/n, NH o/n	(+)	(+)	(+)	(−)	(−)	(−)	(−)	(−)	Profile II	3
	H o/n	(−)	(−)	(−)	(+)	(+)	(+)	(−)	(−)	Profile III	1
	NH o/n	(+)	(+)	(+)	(+)	(+)	(+)	(−)	(−)	Profile V	1
	NH o/n	(−)	(−)	(−)	(−)	(+)	(−)	(+)	(−)	Profile VI	1
	NH o/n	(−)	(−)	(−)	(−)	(−)	(−)	(−)	(+)	Profile VII	1
	Direct plating^a^	(+)	(+)	(+)	(+)	(+)	(+)	(+)	(+)	Profile I	4
PIF		(+)	(+)	(+)	(+)	(+)	(+)	(−)	(+)	Profile VIII	7
Dietary supplements	Direct plating^a^	(−)	(−)	(−)	(+)	(+)	(+)	(−)	(+)	Profile IX	3
		(−)	(−)	(−)	(+)	(−)	(+)	(−)	(+)	Profile X	1
		(+)	(+)	(+)	(+)	(+)	(+)	(+)	(+)	Profile I	3
		(−)	(−)	(−)	(+)	(+)	(+)	(+)	(+)	Profile IV	2
Medicated fish feed	Direct plating^a^	(−)	(−)	(−)	(+)	(+)	(+)	(+)	(+)	Profile IV	7
		(−)	(−)	(−)	(+)	(+)	(+)	(−)	(+)	Profile IX	5
		(−)	(−)	(+)	(+)	(+)	(+)	(−)	(+)	Profile XI	2
		(−)	(−)	(+)	(+)	(+)	(+)	(+)	(+)	Profile XII	7
		(+)	(+)	(+)	(+)	(+)	(+)	(+)	(+)	Profile I	1
		(+)	(−)	(+)	(+)	(+)	(+)	(+)	(+)	Profile XIII	1

(+) = positive by PCR; (−) = negative by PCR; HD = heated, direct plating, NH-D = Not heated, direct plating, H o/n = heated and incubated overnight, NH o/n = not heated and incubated overnight.

a Direct plating on BACARA chromogenic agar (bioMérieux, Hazelwood, MO).

**Table 2:** WGS assembly and annotations of the *B. cereus* strains and reference genomes used in this study.

Source	Biosample sccession	Strain	Assembly name	NCBI GenBank accession	No. of CDS^a^	ST^b^

Spice#	SAMN05415284	MOD1_Bc18	Bc18	GCA_001913615	5654	ND^c^
	SAMN05415285	MOD1_Bc21	Bc21	GCA_001913455	6353	13
	SAMN05415286	MOD1_Bc23	Bc23	GCA_001913405	5729	ND
	SAMN05608038	MOD1_Bc50	Bc50	GCA_001913375	5535	ND
	SAMN05608072	MOD1_Bc55	Bc55	GCA_001913485	5012	1295
	SAMN05608045	MOD1_Bc66	Bc66	GCA_001913385	5568	ND
	SAMN05608063	MOD1_Bc68	Bc68	GCA_001913475	5655	New^d^
	SAMN05608041	MOD1_Bc72	Bc72	GCA_001913395	5473	ND
	SAMN05608061	MOD1_Bc77	Bc77	GCA_001913625	5267	New
	SAMN05608083	MOD1_Bc80	Bc80	GCA_001913315	5538	ND
	SAMN05415288	MOD1_Bc85	Bc85	GCA_001913635	5470	ND
	SAMN05608078	MOD1_Bc89	Bc89	GCA_001913305	5201	ND
	SAMN05608051	MOD1_Bc119	Bc119	GCA_001913295	5359	ND
Medicated animal feed#	SAMN05901947	MOD1_Bc97	Bc97_MED5c	GCA_001982885	6267	869
	SAMN05901945	MOD1_Bc105	Bc105_MED12A	GCA_001982925	5831	205
	SAMN05901944	MOD1_Bc106	Bc106_MED11B	GCA_001982935	5773	ND
	SAMN05901942	MOD1_Bc107	Bc107_MED11C	GCA_001982805	5909	ND
	SAMN05901943	MOD1_Bc110	Bc110_MED29C	GCA_001982815	5689	1065
Hybrid tilapia# (medicated)	SAMN05901949	MOD1_Bc143	Bc143	NCBI is Processing	5919	265
	SAMN05905663	MOD1_Bc149	Bc149	GCA_001982855	5328	ND
	SAMN05901941	MOD1_Bc174	Bc174	GCA_001940365	6268	ND
Dietary supplements#	SAMN05415710	MOD1_Bc133	Bc133	GCA_001913535	5498	New
	SAMN05608070	MOD1_Bc135	Bc135	GCA_001913545	5559	120
	SAMN05608069	MOD1_Bc136	Bc136	GCA_001913645	5485	90
	SAMN05608068	MOD1_Bc137	Bc137	GCA_001913555	6642	90
	SAMN05608066	MOD1_Bc139	Bc139	GCA_001913575	5633	8
	SAMN05608065	MOD1_Bc140	Bc140	GCA_001719025	5474	1084
Powdered infant formula#	SAMN05415728	MOD1_Bc12*	Bc12	GCA_002021385	5698	New
	SAMN05415720	MOD1_Bc67*	Bc67	GCA_001901815	6078	205
	SAMN05415740	MOD1_Bc111*	Bc111	GCA_001901895	5686	32
	SAMN05415756	MOD1_Bc112*	Bc112	GCA_001901905	5628	127
	SAMN05415765	MOD1_Bc113*	Bc113	GCA_001901885	5682	205
NCBI genome groups†	SAMN00715886 SAMN02469355	ATCC 10876 LCT-BC244	Bc10876 Bc244	GCA_000160895 GCA_000256545		
	SAMN02604059	B4264	Bc4264	GCA_000021205		
	SAMN02603340	ATCC 14579	Bc14579	GCA_000007825		
	SAMN02596802	BAG1X1–1	Bcbag1×1_1	GCA_000399005		
	SAMN02596803	BAG1X1–2	Bcbag1×1_2	GCA_000291035		
	SAMN02596813	BAG2X1–3	Bcbag2×1_3	GCA_000291515		
	SAMN02596828	BMG1.7	Bcbmg1	GCA_000399345		
	SAMN03457174	CMCC P0011	Bccmcc	GCA_001635955		
	SAMN03325933	F4429–71	Bcf4429_71	GCA_001044635		
	SAMN00727647	F65185	BcF65185	GCA_000161315		
	SAMN02435890	G9241	BcG9241	GCA_000832805		
	SAMN02604060	G9842	Bcg9842	GCA_000021305		
	SAMN00717290	m1293	Bcm1293	GCA_000003645		
	SAMN00727711	Rock1–15	Bcrock1_15	GCA_000161175		
	SAMN00727632	BDRD-ST24	BcST24	GCA_000161055		
	SAMN02596867	VD140	Bcvd140	GCA_000399545		
	SAMN02596871	VD156	Bcvd156	GCA_000290775		
	SAMN02596873	VD169	Bcvd169	GCA_000290735		
	SAMN02596876	VD200	Bcvd200	GCA_000290715		
	SAMN02596796	VD022	Bcvd22	GCA_000290955		
Dairy isolate genomes‡	SAMN03800014	FSL H7–0926	LOBD01	GCA_001584065		
	SAMN03800017	FSL K6–0040	LOBE01	GCA_001584085		
	SAMN03800019	FSL K6–0067	LOMN01	GCA_001583925		
	SAMN03800021	FSL K6–0073	LOMO01	GCA_001583935		
	SAMN03800022	FSL K6–0267	LOMP01	GCA_001583955		
	SAMN03800024	FSL W8–0003	LOMQ01	GCA_001583705		
	SAMN03800025	FSL W8–0050	LOMR01	GCA_001584005		
	SAMN03800027	FSL W8–0268	LOMS01	GCA_001583745		
	SAMN03800031	FSL W8–0523	LOMV01	GCA_001583765		
	SAMN03800023	FSL M8–0117	LONG01	GCA_001583975		
	SAMN03800032	FSL W8–0640	LOQA01	GCA_001583875		
	SAMN03800033	FSL W8–0824	LOQB01	GCA_001583865		
	SAMN03800035	FSL W8–0767	LOQD01	GCA_001583845		

a CDS Coding DNA Sequences.

b ST Sequence Types, MLST.

c ND Not determined.

All ND isolates are non-typable because of one or more of the following reasons:

i) allele not found in the *B. cereus* MLST database

ii) locus not found in the WGS assembly

iii) nucleotide profile could not be determined for the sequence in the query

iv) alleles not recognized by the *B. cereus* MLST database.

d New ST types are

i) as recognized by the database

ii) alleles in the query sequence determined to be eligible for a ST category by the authors.

* WGS assembly from this work originally reported in Carter et al. Genome Announcements (2017).

† NCBI Genome group strains were obtained from https://www.ncbi.nlm.nih.gov/genome/genomes/157.

‡ Dairy isolate genome IDs were obtained from Kovac et al. BMC Genomics (2016) and downloaded from NCBI.

# The 32 genomes published from this study.
