# Supplementary figures and images for: Analysis of enterotoxigenic Bacillus cereus strains from dried foods using whole genome sequencing, multi-locus sequence analysis and toxin gene prevalence and distribution using endpoint PCR analysis
Source: Int J Food Microbiol. Author manuscript; Available in PMC 2024 Nov 7. (PMC11541649; doi:10.1016/j.ijfoodmicro.2018.06.016)

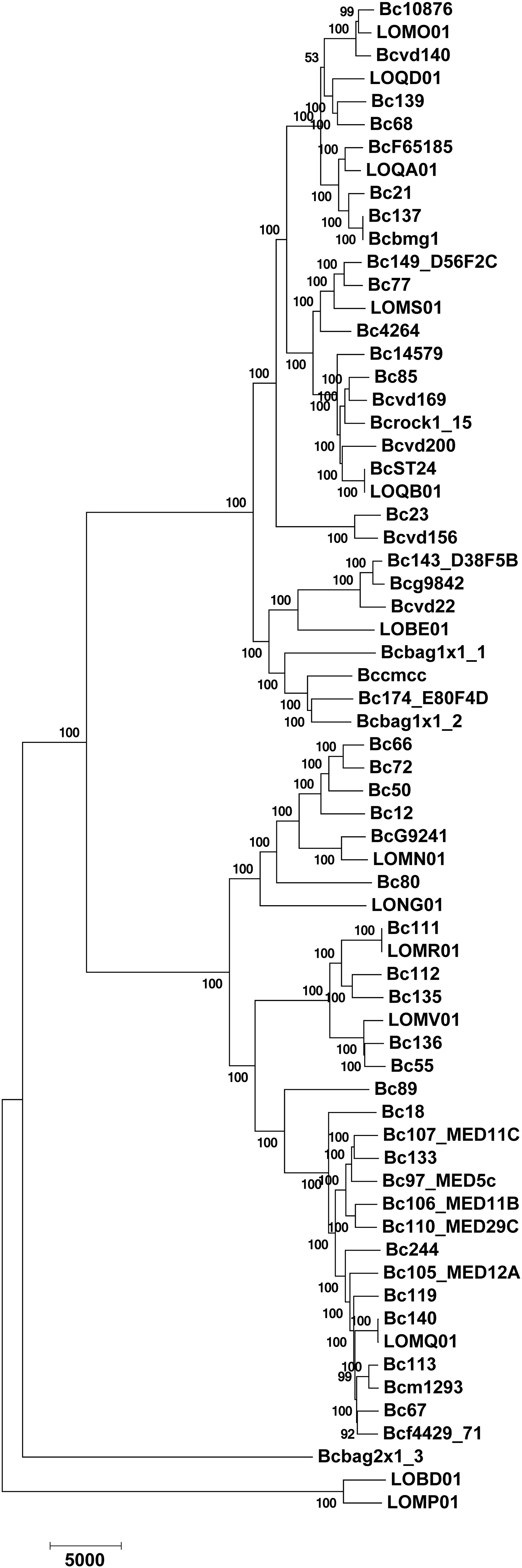

Supplement: Supp fig 1 [file NIHMS2023289-supplement-Supp_fig_1.jpg]

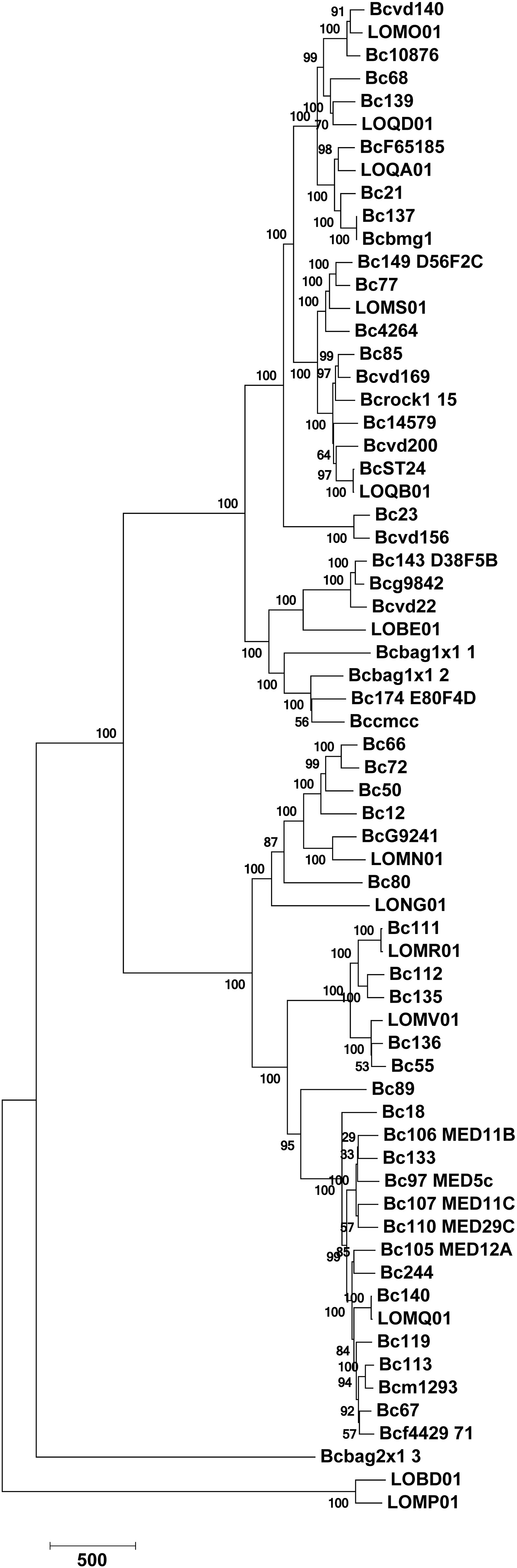

Supplement: Supp Fig 2 [file NIHMS2023289-supplement-Supp_Fig_2.jpg]
